# Necrosis of Femur Resulting in a Malignant Degeneration of the Soft Parts Requiring Amputation

**Published:** 1853-05

**Authors:** C. D. Robinson

**Affiliations:** Almond, Allegany county, N. Y.


ART. IV. — *Necrosis of Femur resulting in a malignant degeneration of the soft parts requiring amputation.* By C. D. Robinson, M. D., of Almond, Allegany county, N. Y.

Prof. F. H. Hamilton:

*Dear Sir,* — Some medical friends consider the case of Necrosis, in part detailed to you, to be worthy of publication, as symptoms of malignancy are extremely rare, or altogether wanting in such cases. With this view, I have thought best to give you a more extended history of the case.

The following is the account as given by the patient:

A. S. S., aged 41 years, a merchant by occupation; of good constitution, of nervo-bilious and sanguine temperament, was attacked with fever when about 11 years of age, which continued some three or four weeks; during convalescence he was taken with pain and swelling of the lower part of the right thigh, which terminated in suppuration, and after some three or four months, pieces of bone made their appearance at the sinus. However, he regained his health so as to engage in the active pursuits of life, occasionally suffering from attacks of inflammation of the thigh, which were frequently followed by the discharge of small spicula of bone. About eight or nine years ago, a large piece made its appearance, which was attached to the shaft of the femur. The patient was often importuned to have this extracted, but refused, as he did not think it necessary, the pain and inconvenience from it being inconsiderable, evidently dreading the pain of extraction.

His general health remained unaffected by the local disease, notwithstanding the profuse discharge, which became very offensive, and the irritation to which he was continually subjected, until about the end of July last. I was now requested to see him. He was affected with violent neuralgic pains in the lumbar and umbilical regions. The limb was swollen and tender to the touch, and the granulations around the old sinus were loose and spongy, like fungus in appearance. Considerable excitement of the circulatory system, loss of appetite and derangement of the liver were present. I attributed the foregoing symptoms to the irritation produced by the loose piece of bone, and urged its removal, to which he consented.

He was put upon the use of conium, quinine, iron, alkalies, and such other remedies as appeared from time to time to be indicated. A slight improvement took place in his general health, though the local symptoms remained nearly the same, except the limb was not as painful.

In Oct. I was obliged to be absent for two weeks, during which he put himself under the care of another practitioner, who induced him to visit New

York, for the purpose of consulting Dr. Mott. Dr. M., together with Dr. Post, pronounced the disease of the soft parts, of a cancroid nature, and recommended amputation. To this Mr. S. did not wish to submit, and soon fell into the hands of one of those irregular practitioners who *boast* of great skill in the cure of old sores. He continued to grow worse, appetite failed, emaciation, loss of sleep from severe pain, colliquative sweats, and irritative fever appeared to be rapidly hurrying him to the grave.

On the 6th of February last, I was again requested to take charge of him. The limb now presented a frightful appearance, being much swollen, and discharging matter from several openings. The different tissues of the thigh were condensed or agglutinated.

I recommended amputation as the only means of saving life, and on the next day, assisted by Drs. Case, Olin, and my partner, Dr. Jas. W. Black, I removed the limb above the junction of the upper with the middle surgical third of the femur. Our patient was put under the influence of chloroform, and bore the operation well, and at the time of writing, has almost entirely recovered; the stump being nearly or quite healed. Constitutional symptoms have all improved since the operation. His appetite is good, and he has nearly regained his ordinary flesh. Countenance looks healthy, and has lost that peculiar sallow expression so characteristic of malignant affections. He is able to go from his dwelling to the store upon crutches.

The bone was found, on examination, carious, for some three inches above the condyles, and so softened that the handle of a scalpel could be pushed, with ease, into its medullary structure.

There were two large openings, one of which extended quite through the bone. I infer from the history of this case, that had the patient suffered the surgeon to have removed the cast-off portion when nature failed, an entire cure would have been effected, and the subsequent malignancy of the soft parts, which extended to the bone itself, was the legitimate effect of long-continued irritation, acting on a susceptible constitution.

Before closing, I will briefly advert to a plan of treatment in necrosis, to which I have resorted in a few cases. It consists in making early and free incisions down to the diseased bone. The great object in the treatment of this disease is to subdue the inflammation before the bone is destroyed by its severity. The usual treatment, as you well know, consists in the exhibition of purgatives and febrifuge medicines, anodynes, with leeches, blisters, fomentations, poultices, &c., at first, and as soon as fluctuation can be detected, an incision, to give free exit to the matter. In the method of treating necrosed bone, to which I have referred, the incision, to be effectual, should be made in the onset of the disease, as the rational end is to relieve the inflamed periosteum and bone before suppuration takes place.

Yours, C. D. ROBINSON.

